# The Green-Eyed Monster: A Brief Exploration of the Jealousy Spectrum

**DOI:** 10.1192/j.eurpsy.2023.2128

**Published:** 2023-07-19

**Authors:** S. Jesus, A. R. Costa, M. Almeida, P. Garrido

**Affiliations:** 1 Departamento de Psiquiatria e Saúde Mental, Centro Hospitalar do Baixo Vouga, Aveiro, Portugal

## Abstract

**Introduction:**

A feeling as ancient as humankind, having been documented in the Bible, represented by mythological figures and appearing as a recurrent theme in art and literature, jealousy is a complex emotion that is non-discriminatory and often associated with negative feelings ranging from insecurity, suspicion, rage, fear to humiliation. Commonly associated with romantic relationships, it typically arises when one perceives a threat, either real or imagined, from a third party in regards to possession or perceived security. Jealousy, like other aspects of the human experience, varies in its expression and intensity, ranging from an adaptive response to a potentially dangerous psychopathological symptom.

**Objectives:**

The authors aim to describe jealousy and discuss the spectrum on which it appears, ranging from an adaptive response to a psychopathological manifestation.

**Methods:**

A brief non-structured literature review was carried out with recourse to various databases such as *Pubmed* as well as complimentary literary sources when deemed pertinent.

**Results:**

Described as a defensive reaction that is expressed as a cognitive, emotional and behavioural response to a perceived threat, jealousy has been discussed in various arenas of thought ranging from evolutionary psychology to philosophy to psychiatry to representation in the arts. It is a difficult term to define as it is a feeling expressed through diverse emotions and behaviours originating from various contexts as well as varying in its intensity. The literature demonstrates that jealousy can exist as an adaptive response, with evolutionary explanations, to a psychopathological expression either as obsessive jealousy or morbid jealousy, also known as Othello’s Syndrome. Each carries its own particularities in terms of expression, clinical significance and intervention. The more often described delusional jealousy, is characterized by the presence of strong, false beliefs that the partner is unfaithful, whereas obsessive jealousy, less commonly described, presents with unpleasant, ego-dystonic and irrational jealous ruminations that the partner could be unfaithful. These thoughts are often accompanied by compulsive verification of the partners’ behaviour. Treatment interventions in these cases are varied and present implications in prognosis.

**Conclusions:**

Jealousy is a complex emotional state and has been described as part of the universal human experience, with research indicating its existence across various cultures. The expression of this emotional experience as well as its potential manifestation types should be taken into consideration by the mental health practitioner when carrying out an evaluation, as treatment interventions and prognosis may vary depending on the presentation.

**Disclosure of Interest:**

None Declared

